# Computerized clinical decision support system for diabetes in primary care does not improve quality of care: a cluster-randomized controlled trial

**DOI:** 10.1186/s13012-019-0955-6

**Published:** 2020-01-07

**Authors:** Annemie Heselmans, Nicolas Delvaux, Annouschka Laenen, Stijn Van de Velde, Dirk Ramaekers, Ilkka Kunnamo, Bert Aertgeerts

**Affiliations:** 1 Department of Public Health and Primary Care, KU Leuven, Kapucijnenvoer 33 blok j, 3000 Leuven, Belgium; 2 Centre for Informed Health Choices, Division for Health Services, Norwegian Institute of Public Health, PO Box 222, Skøyen, 0213 Oslo, Norway; 3 Leuven Institute for Healthcare Policy, KU Leuven, Kapucijnenvoer 35 blok d, 3000 Leuven, Belgium; 4 Duodecim, Scientific Society of Finnish Physicians, PO Box 874, Kaivokatu 10, 00101 Helsinki, Finland

**Keywords:** Decision support systems, clinical, Electronic Health Records, Reminder systems, Diabetes Mellitus, Primary Health Care, Randomized Controlled Trial

## Abstract

**Background:**

The EBMeDS system is the computerized clinical decision support (CCDS) system of EBPNet, a national computerized point-of-care information service in Belgium. There is no clear evidence of more complex CCDS systems to manage chronic diseases in primary care practices (PCPs). The objective of this study was to assess the effectiveness of EBMeDS use in improving diabetes care.

**Methods:**

A cluster-randomized trial with before-and-after measurements was performed in Belgian PCPs over 1 year, from May 2017 to May 2018. We randomly assigned 51 practices to either the intervention group (IG), to receive the EBMeDS system, or to the control group (CG), to receive usual care. Primary and secondary outcomes were the 1-year pre- to post-implementation change in HbA1c, LDL cholesterol, and systolic and diastolic blood pressure. Composite patient and process scores were calculated. A process evaluation was added to the analysis. Results were analyzed at 6 and 12 months. Linear mixed models and logistic regression models based on generalized estimating equations were used where appropriate.

**Results:**

Of the 51 PCPs that were enrolled and randomly assigned (26 PCPs in the CG and 25 in the IG), 29 practices (3815 patients) were analyzed in the study: 2464 patients in the CG and 1351 patients in the IG. No change differences existed between groups in primary or secondary outcomes. Change difference between CG and IG after 1-year follow-up was − 0.09 (95% CI − 0.18; 0.01, *p*-value = 0.06) for HbA1c; 1.76 (95% CI − 0.46; 3.98, *p*-value = 0.12) for LDL cholesterol; and 0.13 (95% CI − 0.91; 1.16, *p*-value = 0.81) and 0.12 (95% CI − 1.25;1.49, *p*-value = 0.86) for systolic and diastolic blood pressure respectively. The odds ratio of the IG versus the CG for the probability of no worsening and improvement was 1.09 (95% CI 0.73; 1.63, *p*-value = 0.67) for the process composite score and 0.74 (95% CI 0.49; 1.12, *p*-value = 0.16) for the composite patient score. All but one physician was satisfied with the EBMeDS system.

**Conclusions:**

The CCDS system EBMeDS did not improve diabetes care in Belgian primary care. The lack of improvement was mainly caused by imperfections in the organizational context of Belgian primary care for chronic disease management and shortcomings in the system requirements for the correct use of the EBMeDS system (e.g., complete structured records). These shortcomings probably caused low-use rates of the system.

**Trial registration:**

ClinicalTrials.gov, NCT01830569, Registered 12 April 2013.

Contributions to the literature

- Computerized clinical decision support (CCDS) systems to manage chronic diseases do not work in a reactive form of healthcare. The right organizational structure must be present if there is to be action, rather than just reaction;
- CCDS systems alone are not a sufficient intensive approach to improving the quality of care in chronic diseases. Multifaceted strategies such as CCDS systems in combination with, e.g. continuing education or feedback mechanisms, organizational changes, and patient-oriented strategies, are probably more appropriate;
- Improvements in outcomes of patients with chronic diseases will only occur if the quality improvement strategy is preceded by fundamental changes in practice design and higher quality of data.

## Introduction

### Background and rationale

Evidence is of no use if it remains buried in the literature and is not implemented in practice. The transformation of evidence from studies into clinical practice guidelines is the first step in this implementation. A computerized clinical decision support (CCDS) system is an information technology-based system designed to improve clinical decision-making [1]. CCDS systems that deliver patient-specific recommendations based on electronic guidelines have shown to successfully deliver the knowledge embedded in evidence-based guidelines [1, 2]. A number of studies have already shown positive findings for some types of decision support systems such as drug-dosing systems and computer-based reminder systems for preventive care services [3–7].

These CCDS systems are particularly important in chronic disease management such as the management of diabetes, which was chosen as the analysis topic of interest in this study. High-quality evidence exists to prevent diabetes complications, but there is still a big gap between the recommended care and the care that patients actually receive [8, 9]. Optimal care of diabetes patients has been difficult to achieve because of the difficulties in sustaining regular monitoring and attention to multiple risk factors over many years [10].

Theoretically, CCDS systems that provide easy access to tailored recommendations and patient information in the Electronic Health Records (EHR) could help physicians to optimize care. However, there is no clear evidence that more complex CCDS systems to manage chronic diseases can enhance patient outcomes or practitioner performance [2, 11, 12].

Only a small majority of CCDS interventions for diabetes management improved practitioner performance. However, the effects are variable, the quality of the evidence is low, and patient-important outcomes were mostly not improved [11–13]. Most trials of CCDS systems in hypertension almost never revealed benefits, and only some showed improvements in the process of care [14, 15]. CCDS systems for asthma and COPD mostly failed to show effectiveness [16, 17]. Furthermore, the reasons for the failure of these systems were not always clear and were rarely explored or reported in previous trials.

Facilitators, barriers, and issues of non-acceptance need to be understood for successful implementation and to minimize unexpected adoption behavior. Electronic systems that are not accepted by their users cannot be expected to be used or even contribute to improving the quality of care. Evidently, earlier studies of CCDS systems with low-use rates could not demonstrate improvements in diabetes care [18, 19]. Studies of CCDS systems with high-use rates have already demonstrated that these systems have a greater chance of success in improving patient and process outcomes [20, 21].

All healthcare professionals in Belgium have free access to an up-to-date database of validated Belgian and nearly 1000 international guidelines, incorporated in a portal that also provides evidence-based medicine (EBM) information from other sources than guidelines (EBPNet), including a CCDS system that is integrated into the EHRs [22–24]. The CCDS system of EBPNet is called the EBMeDS system which was developed by Duodecim [25]. In this study, we assessed the integration of EBMeDS in the software of HealthOne, a Belgian EHR used by many general practitioners. Based on the results of a previous qualitative study in a pilot setting, we adapted the system according to the reflections of the end-users [26], which is an important requirement for the acceptability of the system [27, 28].

The objective of this study was to assess the effectiveness of EBMeDS use for improving diabetes care. This objective was augmented by a formal process evaluation to provide crucial information on the feasibility of using the system in Belgian general practice.

## Methods

### Trial design

A summary of the methods used is provided in this text. We reference a previous publication for the detailed information of the protocol [29].

This study was a cluster-randomized trial with before-and-after measurements in Belgian general practice over 1 year, from May 2017 to May 2018. Patients of 51 primary care practices (PCP), including 120 general practitioners, were randomly assigned by a statistician following simple randomization procedures without any other criteria. The statistician used an electronic random number generator to randomize the PCPs to either the intervention group (IG) or the control group (CG) in a 1:1 ratio, to receive either the EBMeDS reminders or to follow the usual care process. Patients and the statistical analyst were blinded from the intervention; physicians could not be blinded given the nature of the intervention (reminders were displayed on the screens of physicians in the intervention group). All results were analyzed following the intention-to-treat principle.

Trial registration: ClinicalTrials.gov, NCT01830569, Registered 12 April 2013, https://www.clinicaltrials.gov/ct2/show/NCT01830569.

### Setting and participants

All 1630 PCPs using HealthOne (Dutch- and French-speaking) were invited by e-mail to participate. Physicians who did not react to the e-mail after 14 days were sent a reminder. Patients in the care of these PCPs were included in the study regardless of the number of visits during the follow-up period if

1. They were 18 years or older.
2. They had their electronic medical records registered with one of the participating family physicians.
3. They had an established diagnosis of diabetes at the moment of the start of the study (identified as having an International Classification of Primary Care (ICPC-2) code of diabetes type II, a prescription for a diabetes-specific drug, or the necessary laboratory results to confirm diabetes).

### Interventions

#### Trial preparation

To ensure that the point-of-care information provided through the EBMeDS system was context-sensitive, the diabetes-specific reminders and suggestions were adapted to the Belgian context. This adaptation process also included careful selection of the most relevant messages for the Belgian general practitioners, to avoid the risk of alert fatigue.

#### The EBMeDS system in the intervention groups

HealthOne enables physicians to record patient histories and contacts, display test results, access generated notes and reports, and support physicians in decision-making for patient care. The EBMeDS system receives structured patient data from the EHR and matches this data to a knowledge base using software algorithms or scripts. It then returns patient-specific reminders, therapeutic suggestions, and diagnosis-specific guideline links to the user. Electronic forms and calculators (e.g., a calculator for glomerular filtration) are integrated into the system. The decision support is only targeted at the physician and was not designed to be shared with patients.

The EBMeDS system covers a broad spectrum of clinical areas. Reminders are prioritized. Urgent reminders are displayed as pop-ups in red (e.g., high potassium values when taking spironolactone). Reminders that focus on clinical outcomes (such as adding ACE-inhibition in diabetic nephropathy, prescribing aspirin in vascular disease) are indicated in yellow and are placed at the top of the list of reminders. Reminders that are focused on the process or surrogate outcomes (e.g., LDL concentration, HBA1c control) are shown in gray and are situated at the bottom of the list. A screenshot of the interface and a complete list of reminders related to diabetes are displayed in Additional file 1.

Relevant reminders in all clinical areas are shown to the physicians in the intervention group, but the analysis is limited to the treatment and follow-up of diabetes patients.

#### The Evidence Linker in the control and intervention groups

The Evidence Linker is an info-button type of decision support (pull system) that has been integrated into Belgian routine practice since 2012 and is considered part of the usual care process [30]. When entering a diagnosis coded in ICPC-2, relevant clinical practice guidelines can be retrieved by the Evidence Linker and can be consulted on demand by the general practitioner. The Evidence Linker service is offered by the Belgian Centre for Evidence-Based Medicine (CEBAM) and is available to all physicians in the CG as well as in the IG during study follow-up. The content and recommendations of the diabetes guidelines presented in the Evidence Linker are similar to the content of the EBMeDS diabetes reminders. Differences between the intervention and control groups are the method of evidence delivery (“push” versus “pull”) and the format of the recommendations (long guideline format versus short reminder format).

### Outcomes

#### Primary and secondary outcomes

The primary outcome was the 1 year pre- to post-implementation change in glycated hemoglobin (HbA1c). Secondary outcomes were the 1-year differences in LDL cholesterol levels and systolic and diastolic blood pressure. Pre- to post-implementation changes in HbA1c, LDL, and blood pressure were calculated as the difference between the mean value of HbA1c, LDL, and blood pressure of the previous year at the moment of the study start and the mean value of HbA1c, LDL, and blood pressure of the previous year at 6 and 12 months. A composite patient score and a composite process score were calculated representing the change in diabetes control. Each of the parameters in the composite scores was compared with their respective target and each outcome that met the target was assigned points. If all outcomes met the targets, a maximum score of 3 points was assigned to the patient composite score and a maximum score of 9 points was assigned to the process composite score (see Table 1). Primary and secondary outcomes were measured before the start of the study, at 6 months, and at 12 months of follow-up.

**Table 1:** Patient and process composite scores

Variable	Maximum score	Target
HbA1c	2 (4 × 0.5)	0.5 point for each quarter that HbA1c is measured in the previous year
Blood pressure	2 (4 × 0.5)	0.5 point for each quarter that blood pressure is measured in the previous year
LDL cholesterol	1	If LDL cholesterol is at least one time measured in the previous year
Microalbuminuria	1	If microalbuminuria is at least one time measured in the previous year
A prescription of statin	1	If a statin is prescribed in the previous 6 months
A prescription of aspirin/clopidogrel	1	If aspirin or clopidogrel is prescribed in the previous 6 months
A prescription of ACE-inhibitor/sartan if antihypertensive drugs are prescribed	1	If antihypertensive drugs are prescribed in the previous 6 months, ACE-inhibitors or sartans are recommended. If no hypertensive drugs are prescribed, the patient must have a systolic blood pressure < 130 mmHg and a diastolic blood pressure < 80 mmHg to get a score of 1 point. If systolic blood pressure > 130 mmHg or if diastolic blood pressure > 80 mmHg, the patient is diagnosed with hypertension.
Total	9	
HbA1c	1	If mean HbA1c of the previous year < 7%
Blood pressure	1	If mean systolic blood pressure of the previous year < 130 mmHg and mean diastolic blood pressure of the previous year < 80 mmHg
Systolic blood pressure		
Diastolic blood pressure		
LDL cholesterol	1	If mean LDL cholesterol of the previous year < 100 mg/dL
Total	3	

#### Process evaluation

A new record was inserted in the log file for each different decision support (DS) message that was triggered. If the identification (id) code of the DS message already existed in the database, no new record was inserted. The group of DS messages can be classified into four categories: drug contraindications, drug interactions, links to guidelines, and reminders. DS messages were automatically triggered, and users could see that a message was available from their main screen but had to click to view the script content. We did not register the number of clicks on the tab of the decision support (see the “Discussion” section), but rather registered the number of clicks on the menu items or functionalities of the EBMeDS system. The following events were monitored: disabling a reminder, giving feedback about a reminder, giving information about the usefulness of a reminder, and requesting details or information about the reminders.

#### Perceptions

Perceptions and feedback were obtained via structured telephone interviews at the end of the study. One physician from each PCP was questioned. Responses were made as free text on 4 open questions or on a 5-point Likert scale for 11 questions from 1 = “totally disagree” to 5 = “totally agree” (see “Results” section for the content of the questions).

#### Sample size

Based on the data in the literature, a mean difference of 0.3% in HbA1c change could be considered clinically significant (SD of HbA1c change 1.3%) [31, 32]. A sample size of 463 patients in each group, assuming a withdrawal rate of 10%, was necessary to detect a mean difference of 0.3% or an effect size of 0.23 in HbA1c change at four post-treatment occasions, with a two-sided unpaired *t* test, a 5% family-wise significance level and Bonferroni correction for multiple testing, 1:1 allocation and a power of 80%. Additional adjustment for clustering of patients within physician practices by the design effect [33], assuming 10 patients per cluster and an intra-class correlation coefficient of 0.047 based on data in the literature [34], leads to a sample size of 659 patients in each arm.

### Statistical methods

Descriptive statistics are provided regarding baseline variables of the patients and the family physicians, as well as the data of actual use and the answers to the questions concerning perceptions of the EBMeDS system. Intervention and control groups were compared regarding baseline variables to evaluate the randomization.

Differences between intervention and control groups regarding continuous outcome measures (HbA1c, cholesterol levels, and blood pressure) were assessed using linear mixed models (multilevel regression models) with random intercepts for physician practices and the modeling of an unstructured residual variance-covariance matrix to account for repeated measurements over time.

The effect of the intervention on the composite (process or patient) scores was analyzed using proportional odds models for ordinal outcomes. The proportional odds assumption was tested by the score test. Clustering was accounted for by a random effect for physician practices and a random effect for the patient. Analyses were performed using SAS software (version 9.4 of the SAS System for Windows).

## Results

### Baseline measurements

**Fig. 1:**
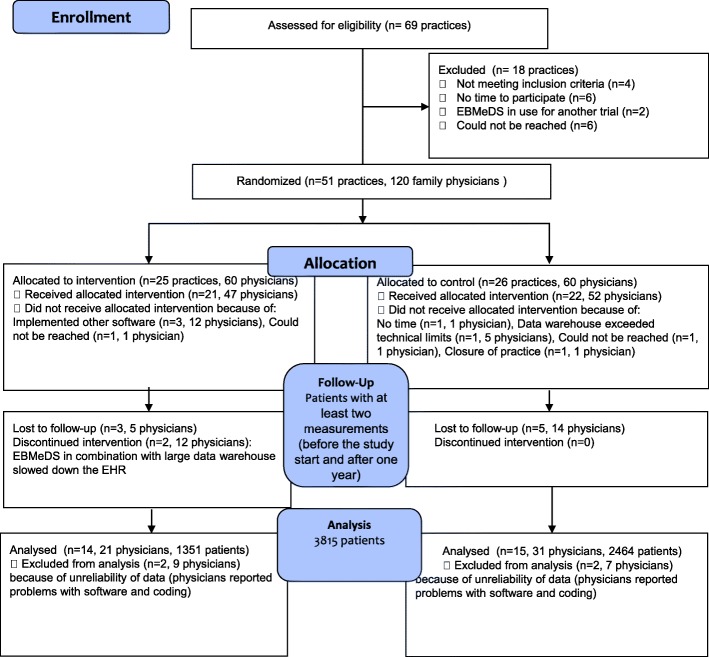
Flow of patients

Of the 69 PCPs screened, 51 PCPs were enrolled and randomly assigned (26 PCPs in the CG and 25 in the IG). Three thousand eight hundred fifteen patients (or 29 practices) were analyzed in the study: 2464 patients in the CG and 1351 patients in the IG (see Fig. 1). Analysis was performed using the intention-to-treat principle, but patients in practices who stopped using the EBMeDS system could no longer be analyzed because these practices also stopped their participation in the trial.

The study population of patients had a mean age of 64.60 years (± 14.65) in the CG and 67.24 years (± 13.31) in the IG. The percentage of females was 51.38% and 47.59%, respectively, in the control and the intervention group. Baseline characteristics between groups were comparable, except for gender, age, number of patients with HbA1c < 7% and number of patients who underwent a yearly micro-albuminuria check. Baseline measures for patients and physicians are displayed in Table 2.

**Table 2:** Baseline measurements of patients and physicians

	Number of (%) of patients *N* = 3815		
	Intervention *n* = 1351	Control *n* = 2464	*p*-value
Sex, females (%)	643 (47.59)	1266 (51.38)	0.025
Age, mean years (SD)	67.24 (±13.31)	64.60 (± 14.65)	< .001
HbA1c, mean % (SD)	7.25 (±1.88)	6.68 (± 1.12)	0.088*
Blood pressure, mean mm Hg (SD)			
Systolic	133.39 (±15.01)	132.93 (± 14.21)	0.34*
Diastolic	77.69 (±7.97)	78.26 (± 19.18)	0.77*
LDL cholesterol, mean mg/dL (SD)	92.16 (±34.12)	97.85 (± 34.68)	0.19*
Number of patients on target for clinical variables			
HbA1c, number of (%)	356/599 (59.43%)	777/1104 (70.38%)	0.03**
Blood pressure, number of (%)	797/1018 (78.29%)	1180/1507 (78.30%)	0.99**
LDL cholesterol, number of (%)	432/672 (64.29%)	850/1475 (57.63%)	0.11**
Number of patients on target for process variables			
Quarterly check HbA1c	28/1351 ( 2.07%)	107/2464 (4.34%)	0.42**
Quarterly check blood pressure	263/1351 (19.47%)	392/2464 (15.91%)	0.59**
Yearly check LDL cholesterol	780/1351 (57.74%)	1489/2464 (60.43%)	0.75**
Yearly check micro-albuminuria	11/1351 (0.81%)	202/2464 (8.20%)	0.03**
Prescription of statin	425/1351 (31.46%)	677/2464 (27.48%)	0.24**
Prescription of aspirin/clopidogrel	44/1351 (3.26%)	57/2464 (2.31%)	0.12**
Prescription of ACE-Inhibitors/sartans if antihypertensive drugs are prescribed	716/1092 (65.57%)	1084/1658 (65.38%)	0.97**
	Number of (%) of physicians *N* = 52		
	Intervention *n* = 21	Control *n* = 31	*p*-value
Sex, females (%)	10/21 (47.62)	10/31 (32.26)	0.38
Age, mean years (SD)	51.19 (± 11.70)	50.94 (± 15.93)	0.83
Language, Dutch (%)	14/21 (66.67)	18/31 (58.06)	0.58

*Baseline differences are tested by means of linear mixed models with a random effect for practice

**Baseline differences are tested by means of logistic regression models with estimation by GEE to account for clustering by practice

### Primary and secondary outcomes

After 1 year follow-up, no significant differences between groups could be found in pre- to post-implementation change in HbA1c, either at 6 months or 12 months follow-up. Mean change of HbA1c after 1 year follow-up was − 0.01 (95% CI − 0.07; 0.05) in the CG and − 0.09 (95% CI − 0.17; − 0.02) in the IG. Change difference between CG and IG was -0.09 (95% CI − 0.18;0.01, *p*-value = 0.06) which is an effect size of 0.10.

Patients in the intervention showed no greater improvements in blood pressure or LDL-cholesterol than patients in the CG. Change difference between IG and CG in LDL-cholesterol after 1 year follow-up was 1.76 (95% CI − 0.46; 3.98, *p*-value = 0.12). Change difference between IG and CG in systolic and diastolic blood pressure after 1 year follow-up was respectively 0.13 (95% CI − 0.91; 1.16, *p*-value = 0.81) and 0.12 (95% CI − 1.25; 1.49, *p*-value = 0.86) (see Table 3).

**Table 3:** Change differences between groups in HbA1c, LDL cholesterol and blood pressure

	Control estimated mean (95% CI)	Intervention estimated mean (95% CI)	Change difference IG-CG estimated mean (95% CI)
HbA1c			
Baseline (BL)	6.69 (6.46; 6.92)	6.99 (6.75; 7.25)	
6 months (6M)	6.64 (6.41; 6.88)	6.97 (6.72; 7.22)	
12 months (12M)	6.68 (6.45; 6.91)	6.89 (6.65; 7.15)	
Change BL–6M	− 0.05 (− 0.08; − 0.00)	− 0.03 (− 0.09; 0.03)	0.02 (− 0.05; 0.09)
Change BL–12M	− 0.01 (− 0.07; 0.05)	− 0.09 (− 0.17; − 0.02)	− 0.09 (− 0.18; 0.01)
Systolic blood pressure			
Baseline	132.40 (129.87; 134.94)	134.41 (131.76; 137.06)	
6 months	131.76 (129.23; 134.30)	134.43 (131.78; 137.07)	
12 months	131.72 (129.19; 134.25)	133.85 (131.21; 136.48)	
Change BL–6M	− 0.64 (− 1.17; − 0.11)	0.02 (− 0.64; 0.68)	0.66 (− 0.19; 1.51)
Change BL–12M	− 0.69 (− 1.35; − 0.03)	- 0.56 (-1.36; 0.24)	0.13 (− 0.91; 1.16)
Diastolic blood pressure			
Baseline	77.87 (75.92; 79.82)	78.08 (76.02; 80.14)	
6 months	77.58 (75.59; 79.57)	77.68 (75.54; 79.81)	
12 months	77.32 (75.49; 79.15)	77.65 (75.77; 79.53)	
Change BL–6M	− 0.29 (− 1.47; 0.88)	− 0.40 (− 1.86;1.06)	− 0.11 (− 1.98;1.76)
Change BL–12M	− 0.55 (− 1.42; 0.32)	− 0.43 (− 1.49; 0.62)	0.12 (− 1.25; 1.49)
LDL cholesterol			
Baseline	100.01 (95.74; 104.27)	93.706 (88.977; 98.435)	
6 months	98.18 (93.91; 102.45)	93.027 (88.314; 97.740)	
12 months	95.92 (91.65; 100.18)	91.375 (86.684; 96.065)	
Change BL–6M	− 1.83 (− 2.71; − 0.95)	− 0.68 (− 1.82; 0.47)	1.15 (− 0.29; 2.59)
Change BL–12M	− 4.09 (− 5.34; − 2.85)	− 2.33 (− 4.17; − 0.49)	1.76 (− 0.46; 3.98)

No intervention effect could be found on change in composite process scores over 6 or 12 months. The probability of improvement in composite process score at 1-year follow-up was 0.43 (95% CI 0.36; 0.51) in the IG versus 0.41 (95% CI 0.34; 0.48) in the CG. Probability of no worsening in composite process score at 1-year follow-up was 0.63 (95% CI 0.55; 0.69) in the IG versus 0.61 (95% CI 0.54; 0.67) in the CG. Odds ratio was 1.09 (95% CI 0.73; 1.63, *p*-value = 0.67).

Composite patient scores over 6 or 12 months were not statistically significantly changed by the intervention. The probability of improvement in the composite patient score at 1-year follow-up was 0.19 (95% CI 0.13; 0.25) in the IG versus 0.24 (95% CI 0.19; 0.29-) in the CG. Probability of no worsening in the composite patient score at 1-year follow-up was 0.87 (95% CI 0.82; 0.91) in the IG versus 0.90 (95% CI 0.87; 0.93) in the CG. Odds ratio was 0.74 (95% CI 0.49; 1.12, *p*-value = 0.16). A listing of the composite scores is provided in Table 4.

**Table 4:** Odds ratio of process and patient composite scores

	Control	Intervention	Odds ratio IG vs CG
Change process score at 6M, number of (%)			
Worsening 6M	445/1314 (33.87%)	309/855 (36.14%)	
No change 6M	320/1314 (24.35%)	206/855 (24.09%)	
Improvement 6M	549/1314 (41.78%)	340/855 (39.77%)	
Change process score at 12M, number of (%)			
Worsening 12M	610/1477 (41.30%)	377/1003 (37.59%)	
No change 12M	224/1477 (15.17%)	166/1003 (16.55%)	
Improvement 12M	643/1477 (43.53%)	460/1003 (45.86%)	
Probability improvement/no worsening process score 6M (95% CI)			
Improvement 6M	0.45 (0.38; 0.52)	0.43 (0.35; 0.51)	
No worsening 6M	0.64 (0.58; 0.71)	0.63 (0.55; 0.69)	0.92 (0.61; 1.39)
Probability improvement/no worsening process score 12M (95% CI)			
Improvement 12M	0.41 (0.34; 0.48)	0.43 (0.36; 0.51)	
No worsening 12M	0.61 (0.54; 0.67)	0.63 (0.55; 0.69)	1.09 (0.73; 1.63)
Change patient score at 6M, number of (%)			
Worsening 6M	49/475 (10.32%)	24/196 (12.24%)	
No change 6M	352/475 (74.11%)	147/196 (75.00%)	
Improvement 6M	74/475 (15.58%)	25/196 (12.76%)	
Change patient score at 12M, number of (%)			
Worsening 12M	60/407 (14.74%)	30/176 (17.05%)	
No change 12M	246/407 (60.44%)	112/176 (63.64%)	
Improvement 12M	101/407 (24.82%)	34/176 (19.32%)	
Probability improvement/no worsening patient score 6M (95% CI)			
Improvement 6M	0.20 (0.16; 0.25)	0.18 (0.13; 0.24)	
No worsening 6M	0.88 (0.85; 0.91)	0.87 (0.81; 0.91)	0.87 (0.59; 1.29)
Probability improvement/no worsening patient score 12M (95% CI)			
Improvement 12M	0.24 (0.19; 0.29)	0.19 (0.13; 0.25)	
No worsening 12M	0.90 (0.87; 0.93)	0.87 (0.82; 0.91)	0.74 (0.49; 1.12)

Post-hoc analysis of the subgroup of patients with a mean HbA1c > 7% at baseline revealed a statistically significant difference in pre- to post-implementation change of HbA1c of − 0.40 (95% CI − 0.70; − 0.09) or an effect size of 0.31 in favor of the intervention group after 12 months of follow-up. Post hoc analyses of the subgroup of patients with LDL > 100 mg/dL and blood pressure > 130/80 mmHg did not show statistically significant differences in pre- to post-implementation change between groups in LDL cholesterol and blood pressure. See Table 5.

**Table 5:** Change differences between groups in a subgroup of patients with a mean HbA1c > 7%, a subgroup of patients with a mean LDL cholesterol > 100 mg/dL and a subgroup of patients with a mean blood pressure > 130/80 mg/dL

	Control Estimated mean (95% CI)	Intervention Estimated mean (95% CI)	Change difference IG-CG Estimated mean (95% CI)
HbA1c > 7%^1^			
Baseline (BL)	7.99 (7.65; 8.33)	8.27 (7.91; 8.63)	
6 months (6M)	7.78 (7.43; 8.14)	8.11 (7.73; 8.49)	
12 months (12M)	7.64 (7.31; 7.98)	7.53 (7.17; 7.89)	
Change BL–6M	− 0.21 (− 0.43; 0.02)	− 0.16 (− 0.42; 0.09)	0.04 (− 0.29;0.38)
Change BL–12M	− 0.35 (− 0.54; − 0.15)	− 0.75 (− 0.98; − 0.51)	− 0.40 (− 0.70; − 0.09)
Systolic blood pressure > 130 mmHg^2^			
Baseline	147.11 (144.67; 149.55)	147.77 (145.10; 150.44)	
6 months	143.75 (141.25; 146.25)	144.94 (142.10; 147.78)	
12 months	141.08 (138.56; 143.60)	141.68 (138.89; 144.47)	
Change BL–6M	− 3.36 (− 4.58; − 2.14)	− 2.83 (− 4.50; − 1.16)	0.53 (− 1.54; 2.60)
Change BL–12M	− 6.03 (-7.49; − 4.57)	− 6.09 (− 7.89; − 4.28)	0.06 (− 2.39; 2.27)
Diastolic blood pressure > 80 mmHg^3^			
Baseline	87.39 (85.85; 88.93)	87.16 (85.47; 88.86)	
6 months	84.00 (82.46; 85.55)	85.11 (83.31; 86.92)	
12 months	82.64 (81.09; 84.19)	83.29 (81.56; 85.02)	
Change BL–6M	− 3.39 (− 4.37; − 2.41)	− 2.05 (− 3.44; − 0.66)	1.34 (− 0.37; 3.04)
Change BL–12M	− 4.75 (− 5.74; − 3.76)	− 3.88 (− 5.09; − 2.66)	0.87 (− 0.69; 2.44)
LDL cholesterol > 100 mg/dL^4^			
Baseline	131.32 (128.29; 134.34)	128.84 (124.92; 132.77)	
6 months	126.88 (123.46; 130.31)	124.40 (119.93; 128.86)	
12 months	118.28 (114.73; 121.82)	115.94 (111.05; 120.82)	
Change BL–6M	− 4.43 (− 6.17; − 2.69)	− 4.44 (− 6.89; − 1.99)	− 0.01 (− 3.01; 2.99)
Change BL–12M	− 13.04 (− 15.64; − 10.44)	− 12.91 (− 17.14; − 8.67)	0.14 (-4.84;5.11)

^1^Subgroup analysis based on 601 patients

^2^Subgroup analysis based on 566 patients

^3^Subgroup analysis based on 566 patients

^4^ Subgroup analysis based on 879 patients

### Process evaluation

Three hundred twenty-two and 354 different DS messages were displayed per practice and per month, respectively, during the first 6 months and between the sixth and the twelfth month of the study. During the first half-year, 9.69% of all the different DS messages were reminders, of which 5.66% were diabetes reminders. During the second half-year, 11.18% of all DS messages were reminders, of which 5.63% were diabetes reminders. Menu items or functionalities of the decision support system (all clinical areas) were used 127 times during the first 6 months and 120 times between the sixth and the twelfth month. The usefulness of the diabetes reminders was indicated four times during the first 6 months. The other menu items were not used within the group of diabetes reminders during the follow-up period of the study.

### Perceptions

Twenty-one physicians were interviewed about their perceptions of the EBMeDS system. Analysis of surveys was limited to the surveys of the physicians whose patients were included in the analysis (14 physicians, see flow chart Fig. 1).

Sixty-four perecent of participants reported that the most important advantage of the EBMeDS system was the possibility to have rapid access to (patient-specific) drug interactions, problems, evidence-based links, etc., without the need for searching. The second most important advantage, reported by 36% of respondents, was the increased alertness by the system. The most important perceived disadvantages of the system were the need to invest time in the system, and the technical problems that occurred (both reported by 29% of respondents). Thirty-six percent of family physicians reported the incorrectness of the reminders (not relevant) as the number one reason for neglecting the reminders, followed by 29% of users who perceived the lack of time as the predominant reason for neglecting reminders.

Only one physician was not satisfied with the EBMeDS system. All physicians (except one who was neutral about the construct) intended to keep using the system in the future, and all physicians (except one) would recommend the system to their colleagues. Only 2 out of 14 physicians did not find the system easy to use. The majority of users (11/14) found that the system worked quickly and that coding within their EHR was easy (9/14). No one found that technical problems caused by the EBMeDS system itself occurred frequently. 9/14 physicians believed that the use of the system could lead to a better quality of care (the other 5 physicians were neutral about the construct). Only 3/14 physicians had the impression that they changed their way of working by using the system. The majority of users (10/14) found that using the system fitted well with the way they liked to work. Half of the physicians (7/14) were neutral about the construct that reminders were relevant; the other half (7/14) were convinced that the reminders were relevant.

## Discussion

### Interpretation of main results

The study showed that the EBMeDS system did not improve diabetes care in Belgian primary care. No significant differences between groups in pre- to post-implementation change of HbA1c, LDL cholesterol or systolic, and diastolic blood pressure could be demonstrated over 6 or 12 months. No intervention effect could be found on change in the composite process and patient scores over 6 or 12 months. Nevertheless, more than 90% of the GPs intend to use the system and would even recommend it to other colleagues.

The effect of computerized clinical decision support systems in the literature varies. CCDS systems in diabetes management may marginally improve clinical outcomes, but confidence in the evidence is low because of the risk of bias [11]. Only a small majority of systems for diabetes in primary care improved practitioner performance, with less success for enhancing patient outcomes [11, 12]. Technologically naïve settings in ambulatory care were mentioned in this review as a possible reason for the failure of these systems [12]. This reason only partly applies to Belgian family medicine, which has been using EHRs since 1980.

Previous studies report that large investments in CCDS systems alone may no longer be warranted [10]. We partly agree with that, but we also believe that it might be possible to benefit from the potential of CCDS with some adaptations.

First, Belgian primary care is organized as a reactive form of healthcare. It is built around the acute care model and is designed to react to an adverse disease, injury, condition or symptom. We studied part of the EBMeDS system that focuses on the management of diabetes, which is a chronic disease. During the short consultation time of a primary care visit, acute care needs often circumvent chronic care and chronic diseases should be organized around the chronic care model [35]. It will be a challenge in the future to transform the daily care of patients with chronic illnesses in family medicine from acute and reactive to proactive and planned healthcare. It is possible that the EBMeDS system alone was not a sufficiently intensive approach. Multifaceted strategies, such as CCDS systems in combination with continuing education and feedback mechanisms, are probably more appropriate in Belgian family medicine at the moment [36, 37]. Studies have already demonstrated the benefits of adding patient-oriented strategies to decision support [12, 38–40].

Second, Belgian primary care is not organized as a uniform system nation-wide and consists mainly of (cooperatives of) individual family physicians, who chose their own software vendor and work together with different laboratories that have their own coding system and measurement units. A lot of these problems could be addressed by the platform-independent, service-oriented architecture (SOA) of the EBMeDS system, which includes a service to normalize data and create the standard variables and objects. However, despite these opportunities within the EBMeDS system, complete records in a structured electronic form remain an important prerequisite for the accuracy and success of the system. Interviews and an exploration of the data of the EHRs taught us that these requirements were not met in all practices.

Third, the level of control of HbA1c at baseline was rather good. But even though mean HbA1c at baseline was 6.68% (± 1.12) in the CG and 7.25% (± 1.88) in the IG, almost 30% of patients in the CG and almost 41% of patients in the IG did not meet the evidence-based target of HbA1c of 7% at baseline. Post-hoc analysis of the subgroup of patients with a mean HbA1c > 7% at baseline revealed a statistically significant difference in pre- to post-implementation change of HbA1c of − 0.40 (95% CI − 0.70; − 0.09) in favor of the intervention group. This result confirms the hypothesis that future studies might better focus on patients with poorly controlled diabetes. Furthermore, only targeting patients with poorly controlled diabetes could reduce the workload resulting from the system, which was indicated by one in three physicians as an important reason for neglecting reminders.

Based on the systematic review of Van de Velde et al., a 16-factor checklist was developed consisting of four domains which may affect the use of the system and the success of the effect of CCDS systems (context, content, system, and implementation) [41]. When evaluating our intervention with the EBMeDS system against the GUIDES checklist, we discovered that improvements could be made in several areas.

Regarding CCDS content, the relevance of reminders could be improved by more accurate coding. 7/14 physicians were neutral about the construct that reminders were relevant, but 5/14 spontaneously reported the lack of relevance as the most important reason for neglecting the reminders. A discussion with general practitioners revealed that the problem of relevance was not related to specific reminders, but to a coding problem in general (e.g. a reminder to measure HbA1c showed up even though it had recently been measured but not correctly captured by the system because of incorrect coding in the EHR). Although we included an ICPC code in the medical records of all patients with an established diagnosis of diabetes before the start of the study, coding has to be a continuous process in the EHR, integrated within the routine clinical practice of physicians and laboratories. The majority of physicians (9/14) reported that they did not have problems with coding in their EHR. However, many of the coding problems that arose in the EHR, e.g. laboratory test codes, were beyond the physicians’ control.

Regarding the CCDS context, physicians confirmed that it was difficult to use or follow the decision support because of the existing workload during consultations. Almost 1 in 3 physicians reported the need to invest time in the system as one of the major disadvantages. Also, 1 in 3 physicians mentioned the lack of time as the most important reason for neglecting reminders. These results were confirmed by our previous EBMeDS study [26] and correspond to the abovementioned difficulties with chronic disease management in an acute care model.

Regarding the CCDS system, the majority of users (8/14) found the system easy to use, which is an important factor in decreasing resistance [42]. Only two respondents reported that the layout for increasing alertness could be better. Technical problems are the most commonly cited reason in the literature for not using the system [43]. Although no physician explicitly agreed with the construct that technical problems frequently arose, technical problems of all kinds (see below), but not related to the EBMeDS system itself, were spontaneously reported by 4/14 of participants as the main disadvantage.

Participants reported they might benefit from a course on coding in the EHR and a more detailed course on the use of EBMeDS. Although each practice received an introduction to the use of EBMeDS and coding before the study started, this apparently did not address the needs of all users.

### Strengths and limitations

An important strength of this study is the adaptation of the EBMeDS system before the study starts to take into account the reflections of users of a previous implementation study in Belgian primary care [26]. The inclusion of end-users’ opinions during implementation formed a strong basis for the acceptability of the system [27, 28, 44].

Second, the addition of qualitative methods to the quantitative methods to evaluate the intervention could offer an important added value in this study. These methods together provided a more thorough evaluation of the intervention than they would have done alone. Furthermore, CCDS systems integrated into the EHR of distributed small PCP have rarely been subjected to rigorous analysis using patient outcomes.

Click events on the decision-support tab could have given us an indication of physician’s interest in using the system. However, the coding of the registration of actual use was imperfect, so that neither the click events nor the number of times a specific reminder was triggered were taken into account. Definite conclusions on actual use of the system could not be drawn which is an important limitation of the study.

We noticed a considerable drop-out rate (43%), mainly because of the following technical problems:

- Software difficulties when exporting the data from the EHRs.
  Data transfer problems were not immediately resolved because of other priorities and accreditation deadlines within the software firm. Communicating the problems to the trusted third party was difficult and delayed the project considerably. During the delay, 3 practices (12 physicians) changed their EHR software and could no longer participate. Due to these problems, we were unable to export the data at the prespecified moments. Files were temporarily saved on the server of the PCPs and were all exported once at the end of the study period, even though we were aware that this procedure posed a risk of data loss. Eight PCPs were lost-to-follow-up mainly for this reason. The planned interim data analysis was not possible because data were not available at interim periods.
- Difficulties with system requirements to activate the data warehouse allowing data analysis.
  Two practices (12 physicians) dropped out of the study because these system requirements significantly slowed down the working of the EHR together with the activation of the EBMeDS system. This problem could not be foreseen because it only occurred in large practices with large data warehouses. These large practices were not pilot-tested. It is important to find a balance between limiting the data sets so that system performance remains optimal, and loading sufficient data so that important data in the patients’ medical history is not lost. On the other hand, the activation of the data warehouse was only required to allow data analysis for the trial. This technical problem will not be relevant when running EBMeDS in practice outside a trial.
- Difficulties with automatic coding of laboratory results.

Four practices (two in the intervention group and two in the control group) were excluded from the final analysis because of problems with the automatic coding of blood results.

Drop-out rates of practices were similar between the IG (44%) and the CG (42%), but relatively larger practices in the intervention group dropped out, resulting in a difference of 10 physicians and 1113 patients between the two groups, with more physicians and patients in the control group (21 vs 31 physicians and 1351 vs 2464 patients). Although the analysis was conducted according to the intention-to-treat principle, patients in these practices could not be analyzed anymore because these practices stopped their participation in the trial.

Evidence of diabetes care changed in the meantime of the delay. At the time of writing the protocol, evidence-based targets in Belgium were HbA1c < 7%, blood pressure < 130/80 mmHg and LDL cholesterol < 100 mg/dL [45]. These were revised in 2015 and evidence-based targets for blood pressure changed to < 140/90 mmHg [46]. Because reminders were consistent with the available recommendations of 2013, results were analyzed using the same evidence-based targets.

The mean follow-up period for each patient was 1 year. This might be too short to detect significant changes in glucose and lipid control. All patients with diabetes type II were included in the study regardless of the number of visits during the follow-up period. Consequently, there might be patients with no second visit, but we assume that these patients were equally distributed between the intervention and control group because of the randomization.

Technical limitations forced us to deviate from protocol. It was not possible to automatically export the necessary variables to calculate cardiovascular risk and nephropathy from the EHR. Due to the amount of time physicians had to spend exporting data, we limited the number of exports to two instead of four.

Possible reasons for low use rates and failure of the system can be summarized as follows:

- The shortcomings in the organizational context of Belgian primary care;
- The lack of complete records in a structured electronic form;
- The fact that there was little room for improvement of HbA1c;
- The lack of relevance of the reminders;
- Possible low use rates of the system;
- Excessive workload during consultations;
- Technical problems that occurred in larger practices;
- The lack of a detailed course on coding in the EHR before the study start;
- Too short follow-up period;

Based on the list above, we concluded that the lack of improvement of the system was mainly caused by imperfections in the organizational context of Belgian primary care for chronic disease management and shortcomings in the system requirements for the correct use of the EBMeDS system (e.g., complete structured records), rather than in the design of the EBMeDS system itself. Only one physician was not satisfied with the EBMeDS system. There were seldom any negative comments during interviews about the design of the EBMeDS system, which leads us to presume that future intervention priorities in Belgium must focus firstly on creating the right conditions for the successful implementation of computerized decision support systems.

### Generalizability

We believe that the way the included practices work is representative of how family medicine is delivered today in Belgium.

Caution is needed when generalizing the results to other countries because of the specific cultural, organizational, and technological infrastructure in Belgium.

## Conclusion

The CCDS system EBMeDS did not improve diabetes care in Belgian primary care. The automatic data export from the EHR posed serious problems. Despite the unsuccessful intervention, the study provided us with important insight into the reasons for the lack of improvement. These consisted mainly of imperfections in the organizational context of Belgian primary care for chronic disease management and shortcomings in the system requirements for the correct use of the EBMeDS system (such as complete structured records), rather than the design of the EBMeDS system itself. National roll-out of EBMeDS can only be successful if combined with the creation of the right conditions for the successful implementation of computerized decision support systems. This implies better data quality (complete records in a structured electronic format and further standardization of coding systems, including laboratory test coding), strategies that contribute to greater use of the system and training initiatives.

It will be a challenge in the future to transform the daily care of patients with chronic illnesses in general practice and the associated technology from reactive to proactive and planned healthcare. It could be that multifaceted strategies, such as continuing education and feedback mechanisms or patient-oriented strategies, are required alongside CCDS systems to optimally implement evidence-based chronic care. It might be a good idea to focus future trials related to computerized decision support systems on patients with poorly controlled diabetes to reduce the workload associated with the system and to follow these patients for a longer period of time.

## Supplementary information

**Additional file 1.** Screenshots of the EBMeDS system and content of the diabetes reminders.

## Data Availability

The datasets used and/or analyzed during the current study are available from the corresponding author on reasonable request.

## Abbreviations

CCDS system: Computerized Clinical Decision Support system
CEBAM: Belgian Centre for Evidence-Based Medicine
CG: Control group
DS: Decision support
EBM: Evidence-based medicine
EHR: Electronic Health Records
HbA1c: Glycated hemoglobin
ICPC: International Classification of Primary Care
ID code: Identification code
IG: Intervention group
PCP: Primary care practice
SD: Standard deviation
