# Supplementary figures and images for: Computerized clinical decision support system for diabetes in primary care does not improve quality of care: a cluster-randomized controlled trial
Source: Implement Sci. 2020 Jan 7;15:5. doi: 10.1186/s13012-019-0955-6 (PMC6947861; doi:10.1186/s13012-019-0955-6)

## Slide 1
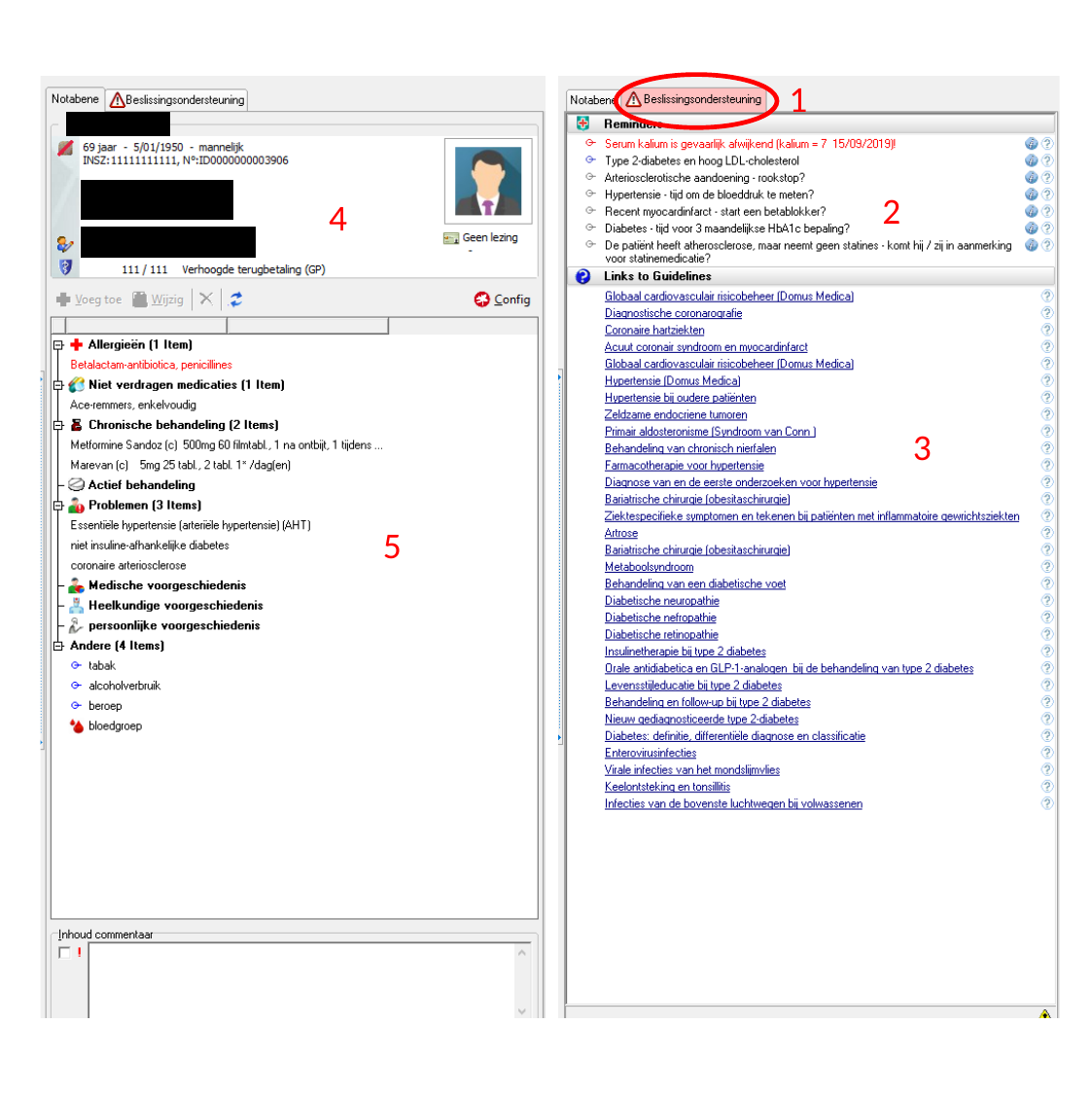

1
2
4
3
5

Supplement: Supplementary file 1 — Additional file 1. Screenshots of the EBMeDS system and content of the diabetes reminders. [file 13012_2019_955_MOESM1_ESM.pptx]
